# Functional analysis of the p.[Arg74Trp;Val201Met;Asp1270Asn]/p.Phe508del *CFTR* mutation genotype in human native colon

**DOI:** 10.1002/mgg3.526

**Published:** 2019-01-01

**Authors:** Sylvia Schucht, Rebecca Minso, Christiane Lex, Jochen Reiss, Frauke Stanke, Stephanie Tamm, Andrea van Barneveld, Burkhard Tümmler

**Affiliations:** ^1^ Pediatric Pulmonary and Allergology Outpatient Clinic, Paediatric Cardiology and Intensive Care Medicine Universitätsmedizin Göttingen Göttingen Germany; ^2^ Clinical Research Group ‘Molecular Pathology of Cystic Fibrosis’, Clinic for Paediatric Pneumology, Allergology and Neonatology Hannover Medical School Hannover Germany; ^3^ Institute for Human Genetics Universitätsmedizin Göttingen Göttingen Germany; ^4^ Biomedical Research in Endstage and Obstructive Lung Disease Hannover (BREATH) Member of the German Center for Lung Research Hannover Germany

**Keywords:** CFTR bioassay, CFTR immunoblot, complex allele, cystic fibrosis, intestinal current measurement

## Abstract

**Background:**

The impact of complex alleles on CFTR processing and function has yet not been investigated in native human tissue.

**Methods:**

Intestinal current measurements (ICM) followed by CFTR immunoblot were performed on rectal biopsies taken from two siblings who are compound heterozygous for the *CFTR *mutations p.Phe508del and the complex allele p.[Arg74Trp;Val201Met;Asp1270Asn].

**Results:**

Normal and subnormal chloride secretory responses in the ICM were associated with normal and fourfold reduced amounts of the mature glycoform band C CFTR, respectively, consistent with the unequal clinical phenotype of the siblings.

**Conclusion:**

The combined use of bioassay and protein analysis is particularly meaningful to resolve the CFTR phenotype of “indeterminate” borderline *CFTR* genotypes on a case‐to‐case basis.

## 1 INTRODUCTION

Cystic fibrosis (CF, OMIM 219700) is caused by mutations in the *CFTR *(GenBank M28668.1) (Elborn, 2016; Ratjen et al., 2015). The CF Mutation Database (http://www.genet.sickkids.on.ca) currently lists more than 2,000 mutations in the *CFTR*, but only a small number of them are clearly defined as CF‐causing based on epidemiological data and functional studies (http://www.cftr2.org) (Sosnay et al., 2013). Genotype–phenotype correlations become even more complicated in case of complex alleles, that is, two or more *CFTR *mutations in cis on the same allele. The first reported case was the revertant mutation p.Arg553Gln which, when carried in cis together with the major mutation p.Phe508del, was associated in the index case with borderline chloride levels in sweat test (Dörk et al., 1991) and which, when expressed in heterologous cells, could partially correct the processing and gating defect caused by the p.Phe508del mutation (Teem et al., 1993). On the other hand, the combination of mutations may be additive on phenotype (Terlizzi et al., 2017) so that the processing and/or function of CFTR become more and more impaired. For example, when expressed in heterologous systems, p.Arg74Trp CFTR behaved like a neutral polymorphism, the double mutant p.[Arg74Trp; Asp1270Asn] exhibited a reduced cAMP‐mediated anion transport and the triple mutant p.[Arg74Trp;Val201Met;Asp1270Asn] was moreover impaired in the posttranslational processing and trafficking of CFTR (Fanen et al., 1999; Terlizzi et al., 2017). The corresponding clinical phenotype, however, is less clear. Individuals with p.Phe508del and p.[Arg74Trp;Val201Met;Asp1270Asn] in trans have been reported to be healthy (Brugnon et al., 2008) or to suffer from a CFTR‐related disorder (CFTR‐RD) or CF (Claustres et al., 2004).

Here, we report on the yet uncharacterized phenotype of this complex *CFTR* allele in patient's tissue in order to unravel of how the *CFTR *mutation genotype translates into basic defect and clinical phenotype in vivo.

## 2 MATERIALS AND METHODS

### 2.1 Subjects

Two siblings compound heterozygous for the *CFTR *mutations p.Phe508del and p.[Arg74Trp;Val201Met;Asp1270Asn] were examined. Their clinical status was determined by height, weight, spirometry, and multiple‐breath nitrogen washout. Mutations in the *CFTR *had been determined in the two index cases and their parents by sequencing of all exons and flanking intron sequences (Stuhrmann‐Spangenberg, Aulehla‐Scholz, Dworniczak, & Reiss, 2009).

### 2.2 Ethical compliance

The study (no. 2771) was approved by the Ethics Committee of Hannover Medical School.

### 2.3 Cell culture

Human CF airway epithelial cells (CFBE41o‐; homozygous for the p.Phe508del mutation) (Kunzelmann et al., 1993) and human non‐CF airway epithelial cells expressing CFTR (16HBE14o‐) (Cozens et al., 1994) were cultured in adherent flasks in MEM medium (Thermo) supplemented with 10% fetal calf serum, 100 U/ml penicillin, and 100 μg/ml streptomycin; at 37°C in a humidified atmosphere of 5% CO_2_/95% O_2_, with medium changes twice weekly. T84 cells were grown as monolayers in DMEM/Ham's F12 (1:1, by vol.) supplemented with 15 mM Hepes, pH 7.5%, and 5% (by vol.) fetal calf serum. Medium was changed every 3 days. Confluent monolayers were subcultured at intervals of 7–9 days.

### 2.4 Intestinal current measurements (ICM)

The electrogenic transport of ions across the intestinal epithelium was measured as short circuit current (*I*_SC_) by ICM following the Standard Operating Procedure (SOP), version 2.7, of the ECFS Diagnostic Network Working Group.

Rectal biopsies were collected with a suction biopsy device (Model SBT‐100, Trewaris Surgical, Bayswater, Australia), immediately stored in tissue medium (medium 199 containing Hank's salts, l‐glutamine, and 25 mM HEPES complemented with 5 mM glycine and 0.5 mM sodium‐dl‐β‐hydroxybutyrate or RPMI‐1640 medium with l‐glutamine and sodium bicarbonate) and mounted in recirculating micro‐Ussing chambers (Physiologic Instruments, San Diego, USA).

The luminal and basolateral compartments were filled with a ${\text{HCO}}_{3}^{-}$ containing buffer of the following composition: 128 mM NaCl, 4.7 mM KCl, 20.2 mM NaHCO_3_, 10 mM HEPES, 0.3 mM Na_2_HPO_4_, 1 mM MgCl_2_, 1.3 mM CaCl_2_, 10 mM d‐glucose. The solution was kept at 37°C and gassed continuously with a mixture of 95% O_2_/5% CO_2_, which maintained the pH at 7.4. Experiments were performed under short circuit conditions, and *I*_SC_ was recorded continuously throughout the experiment.

To determine CFTR Cl^−^ channel function, rectal tissues were equilibrated in Ussing chambers for 40 min in the presence of amiloride (10 µM, luminal) to block electrogenic Na^+ ^absorption and indomethacin (10 µM, basolateral) to inhibit prostaglandin E2 synthesis and endogenous cAMP formation. Previous studies demonstrated that endogenous CFTR activity is largely inhibited under these experimental conditions (Bronsveld et al., 2000). To assess CFTR‐mediated Cl^−^ transport, we next measured lumen‐positive (Cl^− ^secretory) *I*_SC_ responses induced by cAMP‐dependent stimulation with 3‐isobutyl‐1‐methylxanthine (IBMX, 100 μM) and forskolin (1 μM) added to the basolateral compartment. In normal human colon, CFTR‐mediated Cl^‐^ secretion (lumen‐positive *I*_SC_ responses) is augmented by cholinergic co‐activation, which leads to an increase in intracellular Ca^2+^ and stimulation of basolateral Ca^2+^‐dependent K^+^ channels that increase the electrical driving force for luminal Cl^−^ secretion via CFTR (Bronsveld et al., 2000; Roth et al., 2011). In CF colon, cholinergic co‐activation results in an initial inverse lumen‐negative *I*_SC_ response reflecting luminal K^+^ secretion, whereas the lumen‐positive Cl^−^ secretory response is absent or reduced depending on the severity of mutant CFTR malfunction. To increase the driving force for CFTR‐mediated Cl^‐^ transport, rectal tissues were therefore activated with carbachol (CCH; 100 μM, basolateral) in the presence of IBMX and forskolin, and CCH‐induced lumen‐negative (K^+^ secretory) and lumen‐positive (Cl^−^ secretory) *I*_SC_ responses were determined. Thereafter, all non‐CFTR chloride channels were inhibited with 0.2 mM 4,4′‐Diisothiocyano‐2,2′‐stilbenedisulfonic acid (DIDS) followed the addition of 0.5 mM histamine to evoke again a chloride secretory response (Bronsveld et al., 2000).

Measurements were performed in four rectal mucosa biopsy specimens. After ICM, the biopsies were frozen and stored at −80°C until use.

### 2.5 CFTR immunoblot analysis

Biopsies of the elder sibling were sampled and processed in 2012 following the procedure described previously (van Barneveld et al., 2010). Biopsies were homogenized in the presence of 10 mM iodoacetamide, 20 mM PMSF, 20 mg/ml pepstatin and antipain, 100 µg/ml leupeptin and antiprotonin, and 500 µg/ml soybean trypsin‐inhibitor in Tris buffer (20 mM Tris/HCl, 150 mM NaCl, pH 8). The lysis started by incubation with 0.03% SDS for 60 min, followed by 1% (v/v) Triton X‐100% and 0.5% (w/v) sodium deoxycholate for 2 hr. After centrifugation (16,000 *g*, 4°C, 20 min), the supernatant was incubated with the specific pre‐immune serum and protein A‐sepharose for 60 min. Immunoprecipitation (IP) was carried out with the in‐house polyclonal anti‐CFTR antibodies R40, R66, and R16 in the presence of protein A‐ and protein G‐agarose. Pellets were washed several times (van Barneveld et al., 2010). CFTR‐immunoreactive bands were detected on 5% SDS–PAGE separated PVDF‐membranes with mABs 570 and 596 (1:500) in 0.2% I‐Block (Tropix, Applied Biosystems) in 0.05% Tween‐TBS (T‐TBS) and pre‐adsorbed anti‐mouse IgG‐HRP from donkey (1:300,000, Abcam) in 0.2% I‐block in T‐TBS incubating with ECL Advance (GE Healthcare) for 20 s. The CFTR‐immunoreactive signal was quantified by densitometry of Hyperfilms ECL (GE Healthcare) exposed to ECL Advance‐covered immunoblot for 1, 5, 10, 30, and 55 s. As markers for calibration of the CFTR B‐ and C‐bands in rectal biopsies, the B‐ and C‐bands of T84 immunoprecipitates separated on the same gel and the protein marker Precision plus Protein Standards All Blue (10–250 kDa, Bio‐Rad) were used. The amount of protein was determined by the Bradford assay. Biopsies of the younger sibling were sampled and processed in 2017. Frozen biopsies were lysed in 50 µl buffer (50 mM Tris, pH 6.8; 10% glycerol; 0.1 M DTT; 10^−4^ diluted protease inhibitor cocktail [SRE 0055, Sigma]; 2% SDS) supplemented with 0.5 µl PMSF and 0.5 µl 1:20‐diluted Omnicleave endonuclease (Epicentre) for 10 min at room temperature and thereafter for 30 min at 37°C. After removal of insoluble debris by centrifugation, a mixture of 15 µl supernatant/15 µl glycerol was separated at 4°C by 6% SDS–PAGE with 1.5 V/cm for 17 hr and then 9 V/cm for 5 hr (Kälin, Claass, Sommer, Puchelle, & Tümmler, 1999) in a Bio‐Rad Mini‐PROTEAN Tetra Cell. These electrophoresis conditions were chosen to achieve a high resolution of proteins of 100 kDa and larger such as CFTR, albeit all proteins below 70 kDa will be eluted into the lower buffer chamber by iontophoresis. Electrotransfer of within‐gel remaining proteins onto Amersham Protran Supported 0.45 NC membranes was performed for 18 hr at 44 mA and 0°C (Kälin et al., 1999). CFTR immunoreactive bands were detected on the blot by sequential incubation with first anti‐CFTR mAbs 217, 570, 596, 660 (1:1,600 dilution, 4°C, overnight), then secondary goat anti‐mouse IgG (Abcam) (1 hr, room temperature), and finally SuperSignal West Femto Maximum Sensitivity Substrate (Thermo) according to the instructions of the manufacturers.

## 3 CASE REPORT

The currently 8‐year and 5‐year old male index cases are two siblings of German maternal and Moroccan paternal descent who are compound heterozygous for the *CFTR *mutations p.Phe508del on the maternal allele and p.[Arg74Trp;p.Val201Met;p.Asp1270Asn] on the paternal allele. The elder boy suffered from recurrent episodes of obstructive bronchitis and had recurrent detection of *Staphylococcus aureus *and of *Haemophilus influenzae *in respiratory specimens. The younger boy is healthier. He experienced fewer episodes of airway infections. Throat swabs were repeatedly positive for *H. influenzae*, but never for *S. aureus*. Spirometry is normal for age in both siblings. Multiple‐breath nitrogen washout tests (Poncin, Singer, Aubriot, & Lebecque, 2017) yielded slightly elevated lung clearance indices of 8.0 and 7.8 for the elder and younger boy, respectively.

**Figure 1:**
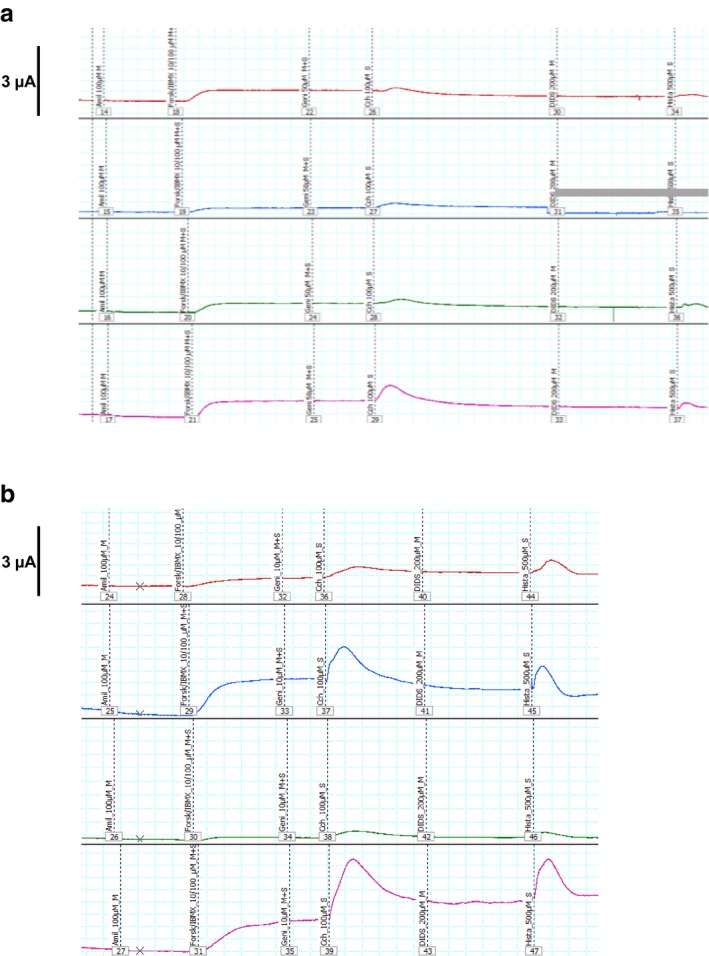
Intestinal current measurements of four rectal suction biopsies collected from (a) the elder (upper panel) and (b) the younger (lower panel) p.Phe508del/p.[Arg74;Val201Met;Asp1270Asn] compound heterozygous siblings. The original tracings show the responses (from left to right) to amiloride, forskolin/IBMX, genistein, carbachol, 4,4′‐Diisothiocyano‐2,2′‐stilbenedisulfonic acid (DIDS), and histamine. Please note that the measurements were performed in Mini‐Ussing chambers with tissue sliders with an aperture area of either 0.018 or 0.011 cm^2^. The registration of the blue tracing in (a) became invalid after exposure to DIDS because of technical reasons (gray bar)

**Figure 2:**
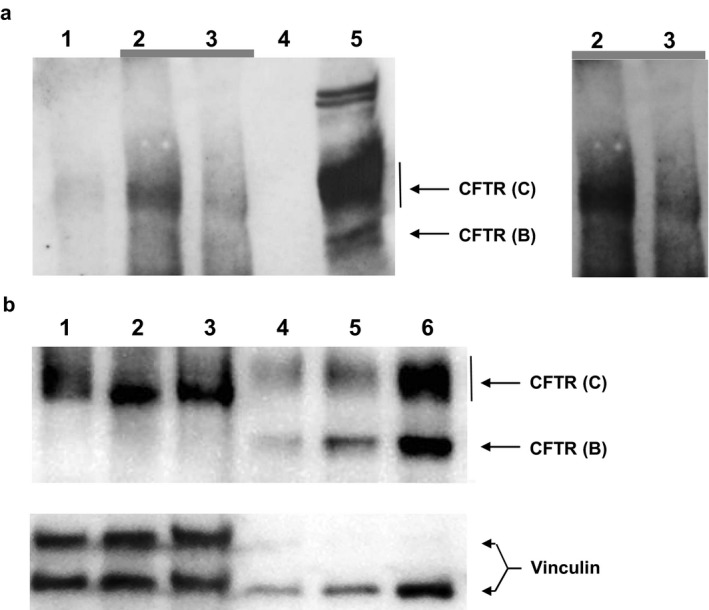
CFTR immunoblot. (a) Immunodetection of CFTR (from left to right) in lysates of rectal suction biopsies by immunoprecipitation (IP) and subsequent immunoblot from a healthy non‐CF subject (lane 2) and its IP bead control (lane 1), the elder p.Phe508del/p.[Arg74;Val201Met;Asp1270Asn] compound heterozygous index case (lane 3) and its IP bead control (lane 4) and in lysates of T84 cells (lane 5). The right panel shows electronically enhanced signals of lanes 2 and 3 of the blot in order to more clearly visualize the CFTR immunoreactive signals of the biopsies. (b) Immunodetection of CFTR by immunoblot of lysates of rectal suction biopsies of two healthy non‐CF subjects (lanes 1, 3; 87 µg each) and of the younger p.Phe508del/p.[Arg74;Val201Met;Asp1270Asn] compound heterozygous index case (lane 2; 87 µg) (lane 1–3) and of non‐CF 16HBE14o cells (lane 4, 15 µg; lane 5, 35 µg; lane 6, 50 µg). Vinculin signals are provided as a western blot loading control

The basic defect was investigated with the CFTR biomarkers sweat chloride concentration in Gibson–Cooke pilocarpine sweat tests and chloride secretory responses in intestinal current measurements (ICM; Figure 1) (De Boeck et al., 2011) followed by immunoblot analysis of CFTR protein (Figure 2) (van Barneveld et al., 2010).

Chloride levels in sweat tests were in the lower intermediate range between 40 and 45 mmol/L in both siblings (Table 1). ICM of rectal biopsies taken from both siblings yielded normal chloride secretory responses to forskolin/IBMX (Table 1). Since p.Phe508del CFTR responses are within the range of a few percent of wild‐type (van Barneveld et al., 2010), the cAMP‐linked chloride secretion can be attributed to the CFTR triple mutant. In contrast, the transient pulses of chloride secretion evoked in the biopsies by basolateral Ca^2+^‐dependent K^+^ efflux induced by carbachol or histamine (Bronsveld et al., 2000) were donor‐dependent either in the normal or in the CF range (Figure 1). The carbachol‐ and the histamine‐mediated responses of the biopsies of the younger sibling were in the normal range, but the responses of the specimens of the elder sibling were in the CF range below the lowermost value ever measured with the same protocol in non‐CF probands (Table 1). The profile of a normal response to forskolin/IBMX and low responses to carbachol and histamine seen in the elder sibling's biopsies is typical for individuals with very mild CF (Stanke et al., 2008) and can be attributed to the drug concentrations selected by the current “SOP” for ICM. Even minute amounts of residual CFTR activity will generate a response to forskolin/IBMX, but the response will saturate at about 20% of wild‐type CFTR activity. Conversely, the chloride secretory responses to carbachol and histamine are approximately proportional to the CFTR activity of the biopsy. In conclusion, the elder sibling exhibited the phenotype of a very mild CF in the ICM, whereas the younger sibling showed a normal response typical for a healthy non‐CF individual.

**Table 1:** Clinical and electrophysiological data of the two boys bearing p.Phe508del and the p.[Arg74;Val201Met;Asp1270Asn] complex allele at the date of intestinal current measurements (ICM) assessment compared to non‐cystic fibrosis (CF) subjects

	Subject 1	Subject 2	Non‐CF (*n* = 68; median [inner quartiles, range])^a^
Current age (months)	108	82	
Age at the date of ICM (months)	36	63	
Age at diagnosis (months)	25	29	
Cause of diagnosis	respiratory	familiarity	
Pancreatic status^b^	PS	PS	
FEV_1 _(% predicted)	109	113	
Lung clearance index	8.0	7.8	
Sweat chloride (mmol/L)	42	45	
ICM (µA/cm^2^): response to			
IBMX/forskolin	37 ± 18	33 ± 25	31 (20–54; 10–104)
Carbachol	14 ± 2	81 ± 46	77 (43–144; 15–250)
Histamine	13 ± 4	49 ± 31	72 (37–125; 14–250)

a Own in‐house data due to the lack of published multicenter reference data for Standard Operating Procedure 2.7.

b Pancreatic status = exocrine pancreatic sufficient.

Next, we determined the CFTR protein levels in the biopsies (Figure 2). The immunoblot detected fourfold lower amounts of complex glycosylated CFTR in immunoprecipitates of rectal biopsies collected from the elder sibling than in specimens taken from wild‐type controls (Figure 2a). In contrast, the intensity of immunoreactive CFTR C‐ and B‐bands was indistinguishable between samples from the younger sibling and non‐CF controls (Figure 2b). Thus, the low and normal responses of the intestinal epithelium to carbachol and histamine observed in the ICM corresponded to low and normal levels of the mature CFTR glycoform, respectively.

## 4 DISCUSSION

The diagnosis of CF is typically based on clinical features, a pathological sweat test and the detection of two disease‐causing mutations in the *CFTR* (Elborn, 2016). The scenario becomes more complex in case of *CFTR *sequence variants that confer substantial residual activity (Bombieri et al., 2011; De Boeck et al., 2011; Sosnay et al., 2017). Subject‐to‐subject variation in phenotype may be so pronounced that the cftr.2 database classifies these sequence variants as “indeterminate” and recommends that “clinical criteria alone should be used whether a person with this variant has CF” (Sosnay et al., 2013). If isolated, all three mutations of the complex allele studied here, p.Arg74Trp, p.Val201Met; p.Asp1270Asn, fall into the category “indeterminate”. Hence, to resolve the impact of the complex allele on CFTR function, we combined the CFTR bioassay ICM with immunochemical CFTR protein analysis. In agreement with the unequal clinical presentation, wild‐type CFTR activity corresponded with wild‐type levels of CFTR protein in the healthier sibling and reduced CFTR activity corresponded with reduced levels of the mature CFTR glycoform in the unhealthier sibling. Immunoblot analysis of CFTR glycoforms in patients’ specimens is technically challenging and has yet been applied to only a few common *CFTR *genotypes causing typical full‐blown CF (Kälin et al., 1999; Kartner, Augustinas, Jensen, Naismith, & Riordan, 1992; van Barneveld et al., 2010). However, as shown in this case report, the combined use of bioassay and protein analysis is particularly meaningful to resolve the individual CFTR phenotype of “indeterminate” borderline *CFTR* genotypes on a case‐to‐case basis.

## CONFLICT OF INTEREST

None declared.
