# Supplementary material for: Functional analysis of the p.[Arg74Trp;Val201Met;Asp1270Asn]/p.Phe508del CFTR mutation genotype in human native colon
Source: Mol Genet Genomic Med. 2019 Jan 1;7(2):e00526. doi: 10.1002/mgg3.526 (PMC6393651; doi:10.1002/mgg3.526)
Supplement: Supplementary file 1 [file MGG3-7-na-s001.doc]

Supporting information

**Material and Methods**

*Subjects*

Two siblings compound heterozygous for the *CFTR* mutations p.Phe508del and p.[Arg74Trp;Val201Met;Asp1270Asn] were examined. Their clinical status was determined by height, weight, spirometry and multiple-breath nitrogen washout. Mutations in the *CFTR* gene had been determined in the two index cases and their parents by sequencing of all exons and flanking intron sequences (Stuhrmann-Spangenberg et al., 2009). The study (no. 2771) was approved by the Ethics Committee of Hannover Medical School.

*Cell culture*

Human CF airway epithelial cells (CFBE41o-; homozygous for the p.Phe508del mutation) (Kunzelmann et al., 1993) and human non-CF airway epithelial cells expressing CFTR (16HBE14o-) (Cozen et al., 1994) were cultured in adherent flasks in MEM medium (Thermo) supplemented with 10% fetal calf serum, 100 U/mL penicillin, and 100 μ g/mL streptomycin; at 37°C in a humidified atmosphere of 5% CO_2_/95% O_2_, with medium changes twice weekly.

*Intestinal current measurements (ICM)*

The electrogenic transport of ions across the intestinal epithelium was measured as short circuit current (I_SC_) by ICM following the Standard Operating Procedure (SOP), version 2.7, of the ECFS Diagnostic Network Working Group.

Rectal biopsies were collected with a suction biopsy device (Model SBT-100, Trewaris Surgical, Bayswater, Australia), immediately stored in tissue medium (medium 199 containing Hank’s salts, L-glutamine and 25 mM HEPES complemented with 5 mM glycine and 0.5 mM sodium-DL-β-hydroxybutyrate or RPMI-1640 medium with L-glutamine and sodium bicarbonate) and mounted in recirculating micro-Ussing chambers (Physiologic Instruments, San Diego, USA).

The luminal and basolateral compartments were filled with a HCO_3_^-^ containing buffer of the following composition: 128 mM NaCl, 4.7 mM KCl, 20.2 mM NaHCO_3_, 10 mM HEPES, 0.3 mM Na_2_HPO_4_, 1 mM MgCl_2_, 1.3 mM CaCl_2_, 10 mM D-glucose. The solution was kept at 37° C and gassed continuously with a mixture of 95% O2 / 5% CO_2_, which maintained the pH at 7.4. Experiments were performed under short circuit conditions and I_sc_ was recorded continuously throughout the experiment.

To determine CFTR Cl^-^ channel function, rectal tissues were equilibrated in Ussing chambers for 40 min in the presence of amiloride (10 µM, luminal) to block electrogenic Na^+^ absorption and indomethacin (10 µM, basolateral) to inhibit prostaglandin E2 synthesis and endogenous cAMP formation. Previous studies demonstrated that endogenous CFTR activity is largely inhibited under these experimental conditions (Bronsveld et al., 2000) To assess CFTR-mediated Cl^-^ transport, we next measured lumen-positive (Cl^−^ secretory) I_sc_ responses induced by cAMP-dependent stimulation with 3-isobutyl-1-methylxanthine (IBMX, 100 μM) and forskolin (1 μM) added to the basolateral compartment. In normal human colon, CFTR-mediated Cl^-^ secretion (lumen-positive I_sc_ responses) is augmented by cholinergic co-activation, which leads to an increase in intracellular Ca^2+^ and stimulation of basolateral Ca^2+^-dependent K^+^ channels that increase the electrical driving force for luminal Cl^-^ secretion via CFTR (Bronsveld et al., 2000; Roth et al., 2011). In CF colon, cholinergic co-activation results in an initial inverse lumen-negative I_sc_ response reflecting luminal K^+^ secretion, whereas the lumen-positive Cl^-^ secretory response is absent or reduced depending on the severity of mutant CFTR malfunction. To increase the driving force for CFTR-mediated Cl^-^ transport, rectal tissues were therefore activated with carbachol (CCH; 100 μM, basolateral) in the presence of IBMX and forskolin, and CCH-induced lumen-negative (K^+^ secretory) and lumen-positive (Cl^−^ secretory) I_sc_ responses were determined.

Measurements were performed in four rectal mucosa biopsy specimens. After ICM the biopsies were frozen and stored at -80°C until use.

*CFTR immunoblot analysis*

Biopsies of the elder sibling were sampled and processed in 2012. Biopsies were homogenized and immunoprecipitated with anti-CFTR polyclonal antibodies in the presence of protein A- and protein G-agarose as described previously (van Barneveld et al., 2010). The washed immunoprecipitate was separated by 5% SDS-PAGE, blotted onto PVDF membranes and then CFTR immunoreactive bands were detected with anti-CFTR mAbs 570 and 596, anti-mouse-IgG-HRP and ECL Advance (van Barneveld et al., 2010). Biopsies of the younger sibling were sampled and processed in 2017. Frozen biopsies were lysed in 50 µl buffer (50 mM Tris, pH 6.8; 10% glycerol; 0.1 M DTT; 10^-4^ diluted protease inhibitor cocktail (SRE 0055, Sigma); 2 % SDS) supplemented with 0.5 µl PMSF and 0.5 µL 1:20-diluted Omnicleave endonuclease (Epicentre) for 10 min at room temperature and thereafter for 30 min at 37°C. After removal of insoluble debris by centrifugation, a mixture of 15 µl supernatant/15 µl glycerol was separated at 4°C by 6% SDS-PAGE with 1.5 V/cm for 17 h and then 9 V/cm for 5 h (Kälin et al., 1999). Electrotransfer of within-gel remaining proteins onto Amersham Protran Supported 0.45 NC membranes was performed for 18 h at 44 mA and 0°C (Kälin et al., 1999). CFTR immunoreactive bands were detected on the blot by sequential incubation with first anti-CFTR mAbs 217, 570, 596, 660 (1:1,600 dilution, 4°C, overnight), then secondary goat anti-mouse IgG (Abcam) (1 h, room temperature) and finally SuperSignal West Femto Maximum Sensitivity Substrate (Thermo) according to the instructions of the manufacturers.

**References**

Bronsveld, I., Mekus, F., Bijman , J., Ballmann, M., Greipel, J., Hundrieser, J., … , Veeze, H.J. (2000). Residual chloride secretion in intestinal tissue of deltaF508 homozygous twins and siblings with cystic fibrosis. The European CF Twin and Sibling Study Consortium. *Gastroenterology, 119*, 32-40.

Cozens, A.L., Yezzi, M.J., Kunzelmann, K., Ohrui, T., Chin, L., Eng, K., …, Gruenert, D.C. (1994). CFTR expression and chloride secretion in polarized immortal human bronchial epithelial cells. *American Journal of Respiratory Cell and Molecular Biology, 10*, 38-47.

Kälin, N., Claass, A., Sommer, M., Puchelle, E., & Tümmler, B. (1999). DeltaF508 CFTR protein expression in tissues from patients with cystic fibrosis. *Journal of Clinical Investigation, 103*, 1379-1389.

Kunzelmann, K., Schwiebert, E.M., Zeitlin, P.L., Kuo, W.L., Stanton, B.A., & Gruenert, D.C. (1993). An immortalized cystic fibrosis tracheal epithelial cell line homozygous for the delta F508 CFTR mutation. *American Journal of Respiratory Cell and Molecular Biology, 8,* 522-529.

Roth, E.K., Hirtz, S., Duerr, J., Wenning, D., Eichler, I., Seydewitz, H.H., …, Mall, M.A. (2011). The K+ channel opener 1-EBIO potentiates residual function of mutant CFTR in rectal biopsies from cystic fibrosis patients. *PLoS One, 6*, e24445.

Stuhrmann-Spangenberg, M., Aulehla-Scholz, C., Dworniczak, B., Reiss, J. (2009). Leitlinie zur molekulargenetischen Diagnostik der Cystischen Fibrose. *Medizinische Genetik, 21,* 268-275.

van Barneveld, A., Stanke, F., Tamm, S., Siebert, B., Brandes, G., Derichs, N., … , Tümmler, B. (2010). Functional analysis of F508del CFTR in native human colon. *Biochimica Biophysica Acta, 1802,* 1062-1069.
